# Epidemiology and Associated Factors in Transfusion Management in Intensive Care Unit

**DOI:** 10.3390/jcm11123532

**Published:** 2022-06-20

**Authors:** Raúl Juárez-Vela, Eva María Andrés-Esteban, Ivan Santolalla-Arnedo, Regina Ruiz de Viñaspre-Hernández, Carmen Benito-Puncel, Ainhoa Serrano-Lázaro, Pilar Marcos-Neira, Alba López-Fernández, Clara Isabel Tejada-Garrido, Juan Luis Sánchez-González, Manuel Quintana-Díaz, José Antonio García-Erce

**Affiliations:** 1 Doctoral Programme in Medicine and Surgery, Faculty of Medicine, Autonomous University of Madrid, 28049 Madrid, Spain; raul.juarez@unirioja.es; 2 GRUPAC, Department of Nursing, University of La Rioja, 26004 Logroño, Spain; ivan.santolalla@unirioja.es (I.S.-A.); reruizde@unirioja.es (R.R.d.V.-H.); 3 Research Institute IdiPaz, 28029 Madrid, Spain; e.andres@live.com; 4 Department of Business Economics and Applied Economy, Faculty of Legal and Economic Sciences, Rey Juan Carlos University, 28933 Madrid, Spain; 5 Intensive Care Unit, Hospital of Guadalajara, 19002 Guadalajara, Spain; cbenitopuncel@yahoo.com; 6 Intensive Care Unit, Clinic Hospital of Valencia, 46010 Valencia, Spain; aserranolazaro@gmail.com; 7 Intensive Care Unit, Hospital Germans Trias i Pujol, 08916 Badalona, Spain; mpmarcosn@gmail.com; 8 Intensive Care Unit, University Hospital of La Paz, 28046 Madrid, Spain; albalopf@gmail.com; 9 Department of Nursing and Physiotherapy, University of Salamanca, 37007 Salamanca, Spain; juanluissanchez@usal.es; 10 Bank of Blood and Tissue of Navarra, Government of Navarra, 31008 Pamplona, Spain; jagarciaerce@gmail.com

**Keywords:** transfusion, haemoglobin, management

## Abstract

Severe traumatic injury is one of the main global health issues which annually causes more than 5.8 million worldwide deaths. Uncontrolled haemorrhage is the main avoidable cause of death among severely injured individuals. Management of trauma patients is the greatest challenge in trauma emergency care, and its proper diagnosis and early management of bleeding trauma patients, including blood transfusion, are critical for patient outcomes. Aim: We aimed to describe the epidemiology of transfusion practices in severe trauma patients admitted into Spanish Intensive Care Units. Material and Methods: We performed a multicenter cross-sectional study in 111 Intensive Care Units across Spain. Adult patients with moderate or severe trauma were eligible. Distribution of frequencies was used for qualitative variables and the mean, with its 95% CI, for quantitative variables. Transfusion programmes, the number of transfusions performed, and the blood component transfused were recorded. Demographic variables, mortality rate, hospital stay, SOFA-score and haemoglobin levels were also gathered. Results: We obtained results from 109 patients. The most transfused blood component was packet red blood cells with 93.8% of total transfusions versus 43.8% of platelets and 37.5% of fresh plasma. The main criteria for transfusion were analytical criteria (43.75%), and acute anaemia with shock (18.75%) and without haemodynamic impact (18.75%). Conclusion: Clinical practice shows a ratio of red blood cells, platelets, and Fresh Frozen Plasma (FFP) of 2:1:1. It is necessary to implement Massive Transfusion Protocols as they appear to improve outcomes. Our study suggests that transfusion of RBC, platelets and FFP in a 2:1:1 ratio could be beneficial for trauma patients.

## 1. Introduction

Serious traumatic injuries cause more than 5 million deaths a year worldwide, standing out as one of the main health problems that require effective preventive and interventional actions [1,2]. The prevalence of serious trauma patient admission to Intensive Care Units (ICU) is high, with very high-cost estimates [3]. The latest data from the Centers for Disease Control and Prevention of the United States (USA), published in 2020, estimate the cost of these fatal traumatic injuries at 214,000 million dollars, and the cost of non-fatal injuries at 457,000 million dollars [4]. It is estimated that in the USA, annual ICU admissions to the due to traumatic injuries are around 3.3 cases per 100,000 inhabitants, with the risk being greater for men than for women [5]. In Spain, serious trauma injuries account for 39.4% of deaths in people between 15 and 39 years of age, and a pilot study reported a mortality rate of 12% in patients with serious trauma admitted to a hospital ICU [6]. These figures increase progressively throughout Europe; accurate response and appropriate clinical management of patients with severe traumatic injuries represent great challenges for health systems [1].

It is estimated that more than 15% of deaths related to serious traumatic injuries are preventable. Uncontrolled bleeding is the leading cause of preventable death [7,8,9]; and together with it, acute traumatic coagulopathy, which is present in more than 30% of haemorrhagic patients who come to the emergency department after a serious traumatic injury, increases the risk of death [1,10]. The clinical approach to these patients focuses on damage control, combining haemorrhage management, coagulopathy limitation, permissive hypotension, and haemostatic resuscitation [11]. Regarding this bleeding control, different strategies have been shown to be effective; timely recognition of bleeding and its proper management—including blood transfusion—are essential to improve patient outcomes.

The World Health Organization considers transfusion therapy as one of the modern types of health care, stating that its use through evidence-based protocols can save lives; although, knowing its complications, its inappropriate use or use not adjusted to protocols must be avoided [12]. Blood component transfusion protocols have varied in recent years and continue to evolve [13]. The professionals who manage these protocols must consider both that blood products are a scarce commodity, so their efficient use is essential, and that their inappropriate use can make blood transfusion ineffective, even increasing patient morbidity. Among the associated risks that can affect patient safety, circulatory overload, immunomodulation, acute lung injury and infections stand out [14,15]. Changes in the health of the population, such as increased chronicity, comorbidity associated with age, increased life expectancy with the consequent aging of the population, frail patients [16], and a high use in these patients of antiaggregating and anticoagulant drugs, may represent an increased risk as well as use of transfusion therapies [17,18].

The most widely used transfusion threshold to start a blood product transfusion protocol continues to be the blood haemoglobin (Hb) level [19,20]. It is unknown if there are differences in Intensive Care Units (ICU) between patients with severe traumatic injury who receive transfusion, the criteria for transfusion, and improvement in haemoglobin (Hb) levels [17]. Different studies denote the importance of basing decision-making in transfusion medicine not only on the physiological factors that trigger the transfusion, but also on the recognition of symptoms and the clinical status of patients [9,21,22]. Furthermore, early recognition of the need for massive transfusion (≥10 units of red blood cells in 24 h) and activation of massive transfusion protocols (MTPs) have been shown to improve survival in patients with severe traumatic injuries [23,24,25].

The above emphasises the importance of developing multidisciplinary protocols to optimise the transfusion of blood components [26,27]. Adequate management of transfusion therapy is a strategy that represents a challenge for the professionals involved in the treatment of patients with serious traumatic injuries who are admitted to Intensive Care Units; its implementation has proven to be effective in reducing unnecessary blood transfusions and related costs, as well as improving patient safety [28,29]. However, more studies are needed to help us make decisions. Therefore, this study aimed to describe the transfusion management of these patients in Intensive Care Units in Spain, identify factors associated with transfusion, and analyse changes after transfusion therapy, verifying differences between polytraumatized patients with severe trauma injuries and the rest of the patients admitted to ICUs.

## 2. Materials and Methods

### 2.1. Design

An observational cross-sectional-study was conducted. Blood transfusion practices during the first 24 h of patient admission to intensive care units were recorded, selecting one day, namely 12 November 2019, in honour of Dr Bethune, who developed a mobile blood transfusion service in the early 20th century.

### 2.2. Sample

We evaluated 1439 subjects, who were recruited from 111 Spanish ICUs. These subjects were divided into two groups according to type of admission, thus creating a group of polytrauma patients and a second group of patients whose admission to the ICU was caused by other reasons. 109 patients had severe trauma, fulfilling the following inclusion criteria: adult patients (aged ≥ 18 years old) with a moderate or severe trauma injury defined as ISS > 16 points were eligible. A total of 46 patients in the polytrauma group had a diagnosis on arrival at the emergency department that was severe and 30 of them were potentially severe. The most frequent mechanism of injury was impact 30/109, and 30/109 traffic accidents, and the area of injury was craniocerebral trauma, found in 39/109 patients, and thoracic trauma in 16/109.

A total of 1330 patients were admitted to the ICU for other circumstances, 902 (67.82%) for medical reasons and 411 (30.90%) for scheduled or urgent surgery. There were 17 (1.38%) patients with another type of unspecified admission. In any case, patients over 17 years of age admitted to the ICU for reasons other than polytrauma were selected.

Information was collected on demographic variables, such as age, gender (men/women), ICU treatment and follow-up: sequential organ failure assessment (SOFA) score, ICU stay (in days), mechanical ventilation (yes/no), extrarenal purification (yes/no), anticoagulation treatment (yes/no), platelet anti-aggregation therapy (yes/no) and extracorporeal membrane oxygenation (ECMO) (yes/no), indication for transfusion, blood component transfused, number of transfusion events, blood transfusion follow-up (in hours) and haemoglobin, platelets, INR, and MCV before and after transfusion.

### 2.3. Statistical Analysis

The overall incident proportion of patients with severe trauma injury that received at least one red blood cell (RBC) transfusion was recorded.

Frequency distributions were used to describe qualitative variables, whereas means with standard deviation (SD) or medians with interquartile interval (IQI), were used to describe quantitative variables. The comparison between groups was performed using the Chi-squared test; however, to compare non-parametric (qualitative vs. quantitative) variables, the Mann–Whitney *U* test was used.

Multivariate analysis was carried out—using binary logistic regression with the forward conditional method—on the variables that gave statistically significant results in the bivariate analysis, or that could have had a plausible clinical implication. Transfusion was considered the dependent variable (bivariate variable, yes/no). Model calibration was performed using the Hosmer–Lemeshow statistic. The discriminatory power was assessed using the area under the ROC curve (receiver-operator characteristics) obtained by analysing the probability of the value predicted by the multivariate model. The results of the multivariable model were presented as odds ratio with a 95% CI.

Statistical analysis was performed using STATA/SE (College Station, TX, USA) version 16.0 and statistical significance was set at alpha level with *p* = 0.050.

## 3. Results

### 3.1. Patient Characteristics

The mean age was 41.42 ± 18.82 years in polytraumatic patients and most of the patients were men (93) [86%]; however, in the non-trauma group, the mean age was 41.42 ± 18.82 and men represented 64.56%, as shown in Table 1. The difference was statistically significant in both cases. There were no differences in the SOFA severity score or days of stay, but there were differences in the treatment of the two groups during ICU stay. Thus, the incidence of mechanical ventilation was higher in polytraumatised patients (58.72% vs. 46.12%, *p*-value = 0.012) and extrarenal purification was more incident in non-polytraumatised patients (8.96% vs. 1.83%, *p*-value = 0.010). Trauma patients had a higher prevalence of anticoagulation and antiaggregating treatments and the transfusion rate in the first 24 h was higher in polytrauma patients (15.60% vs. 9.50%, *p*-value = 0.041).

### 3.2. Characteristics of the ICUs

Table 2 shows the main characteristics of the ICUs that participated in this study; 98% of them were from public hospitals, whose number of beds was very heterogeneous. It should be noted that 78% of ICUs had an MTP, 61% had a transfusion protocol, and only 35% had a PBM programme.

### 3.3. Clinical Transfusion Practice

Blood was transfused to seventeen patients; the estimated incidence of transfusion was 15.60% (95% CI: 9.22–22.90%) in the group of polytraumatised patients, and 9.50% (95% CI: 7.95–11.17%) in the group of non-polytraumatised patients. None of the characteristics studied differentiated the transfused patients of the two groups except for age, as shown in Table 3 below. The patients admitted to the ICU due to polytrauma had a mean age of 39.00 ± 19.67 compared to the group of non-polytrauma patients who were much older, namely 66.00 ± 14.37, the difference being significant, *p*-value < 0.001.

Most of the indications for transfusion were based on haemoglobin levels, followed by anaemia, not finding a statistical difference between the indication for transfusion on polytraumatic patients and non-polytraumatic patients. The blood transfusion policy was similar in both groups, with a 2:1:1 blood component transfusion policy as shown in Table 4.

There were no differences between the pre-post values of haemoglobin, MCV, international normalised ratio (INR), and platelets in the transfused patients from the polytrauma group; however, we did find differences in haemoglobin and MCV levels in the non-polytraumatized patients (Table 5).

The logistic regression models showed that the factor that mostly influenced the decision to transfuse were haemoglobin levels; thus, lower levels of OR indicated a greater risk of transfusion (Table 5 and Table 6). A patient admitted to the ICU with polytrauma has a 75% higher risk of receiving a transfusion during the first 24 h than a patient admitted for other reasons (OR = 1.75). Other risk factors were also associated with transfusion, such as the SOFA severity scale, where patients with a higher score have a higher risk of receiving a transfusion (OR = 1.13). In addition, being an anticoagulated patient or receiving mechanical ventilation was associated with transfusion, as shown in Table 5.

## 4. Discussion

The present study analyzed the transfusion practices performed in 111 Spanish ICUs. Our results showed that most hospitals had PTMs (78%), which has been associated with a reduction in trauma-related mortality and the overall use of blood products, both in the emergency department and after a period of 24 h [24,25,26,27,28]. Our study shows that the most frequent reason for deciding on transfusion was analytical criteria (34.5% of transfusion events), followed by acute anaemia with shock (31%) and without haemodynamic repercussions (20%). Rapid bleeding control can improve survival for patients with severe traumatic injuries and life-threatening bleeding [5]. A remarkable finding is that 61% of the hospitals had transfusion protocols and only 35% had a PBM programme. These results suggest that the transfusion decision-making of hospitals that had not implemented transfusion protocols may have been more influenced by analytical values, the Hb value, than by the patients’ clinical situation. However, the aetiology of anaemia in critically ill patients may be the inflammatory process and, consequently, the haemoglobin concentration would not consistently reflect the mass of red blood cells [29,30]; thus, the recommendation is not to transfuse solely based on analytical criteria if these are not based on the patient’s clinical status [31]. On the other hand, it was surprising that, although the benefits of PBM programmes have been previously demonstrated [22,32,33], a low proportion of hospitals had implemented this strategy.

In our study, the proportion of transfused patients was 14.82%, very similar to the 15% reported in an observational study of 375,478 ICU episodes conducted in the United States [34] and lower than that reported in another study suggesting that 25% of injured patients admitted to an ICU receive at least one RBC unit, and 2–3% of them receive a massive transfusion [23]. The most transfused blood component was packed red blood cells (94% of total transfusions), followed by platelets (44%), and fresh frozen plasma (FFP) (38%), with a ratio of 2:1:1. It is suggested that platelets, red blood cells, and PFC, be transfused with a 1:1:1 ratio in critically injured patients. However, the optimal ratio remains controversial, and practice varies from centre to centre [25]. For example, Holcomb et al. found no significant difference in mortality at 24 h or 30 days after comparing transfusion ratios of 1:1:1 and 2:1:1 between patients with major trauma and major bleeding [35]. Therefore, the currently available evidence is limited as regards recommending one ratio over the other [33]. Finally, although the demographic and clinical characteristics of polytraumatised and non-polytraumatised patients admitted to ICUs present significant differences, these differences disappear when only transfused patients are analysed, except with regard to the significantly younger age of the polytraumatised patients. Similarly, the practice of transfusion does not show differences between the groups of patients, either in the reasons for the transfusion or in the type of preparation transfused, the number of transfusions, or transfusion control.

On the other hand, it was found that the percentage of transfused men was higher than of women (10.3% vs. 7.4%). This higher prevalence of transfusions in men was observed in both groups of patients, polytraumatized and non-polytraumatized, with no significant differences between them. Gender disparities related to transfusion decision-making in trauma patients are not new; previous studies have addressed transfusion disparities between men and women [36]. Furthermore, despite the different coagulation profiles between genders, current transfusion guidelines are not gender-specific and instead are designed primarily based on the physiological characteristics of men [37,38]. However, our results, in which men are more transfused, should be further analyzed in studies with a longitudinal approach and could be interpreted by way of ICU admission due to trauma. According to our data, the factors that best explain the Spanish ICUs’ clinical decision to administer transfusion include if a patient is polytraumatized and/or being treated with anticoagulants, increased risk of multi-organ failure on the SOFA scale, the need for mechanical ventilation, and a lower Hb value before transfusion. These data corroborate the information provided in other studies that transfusion risk increases in critically ill patients, with a more severe illness and lower haemoglobin level [39].

Trauma is the situation that most often leads to massive haemorrhage, with about 40% of trauma-related mortality due to uncontrolled haemorrhage [40]. To maintain oxygen supply to tissues and restore efficient coagulation, early administration of red blood cells and fresh frozen plasma is recommended [41]. Mechanical ventilation is also associated with transfusion (OR: 1.58; 95% CI: 1.03–2.43). In theory, transfusion could improve oxygen-carrying capacity. However, retrospective reports have shown a positive correlation with prolonged mechanical ventilation, higher mortality rates, nosocomial infections, and worse weaning outcomes [42,43,44]. Regarding the taking of anticoagulants, this group of drugs increases the risk of active bleeding and lower Hb values, which requires a readjustment in the medication of these patients and justifies a higher frequency of transfusion events [45]. On the other hand, anaemia is common in patients admitted to intensive care units. Up to two-thirds of patients admitted to an ICU have anaemia on admission, and the prevalence reaches up to 95% on the third day of stay in the ICU. Anaemia is an independent risk factor for morbidity and mortality during hospital stay [46]. A study of critically ill patients admitted to 139 US hospitals showed that anaemia multiplies the risk of transfusion by more than 2 times [47]. Therefore, the transfusion of red blood cells in all cases, including polytrauma, mechanical ventilation, taking anticoagulants, and anaemia, could have indicated the severity of the disease [44]. This could be supported by the fact that a higher risk of multiple organ failure, measured with SOFA, increased, in this study, the probability of transfusing (OR: 1.13: 95% CI: 1.03–2.44).

It is interesting to observe at the analytical level the different impact that the transfusion produces between the two groups of patients. Non-polytraumatized patients had lower pre-transfusion Hb values than trauma patients (median: 7.6 g/dL vs. 8.6 g/dL), and when measuring post-transfusion Hb, it was found that these patients had significantly increased Hb levels whereas the polytraumatized patients showed smaller, non-significant increases (median value increases: 1.4 g/dL vs. 1 g/dL). These values are in agreement with the literature: it is estimated that the increase in haemoglobin concentration after the transfusion of a unit of red blood cells is approximately 1 g/dL, but it depends on the volume of the unit of red blood cells and the clinic. In addition, factors such as body mass index, sex, haemoglobin value prior to transfusion or continuous blood loss and diuresis secondary to fluid replacement can modify the Hb level increase [48]. On the other hand, guided exclusively by the pre-transfusion Hb values, we can think that some of the polytraumatized patients with Hb values above the 75th percentile (Hb > 10.4) could not have received a transfusion if the hospital in which they were treated had had a PBM programme. Most transfusion guidelines recommend restrictive transfusion with an Hb cut-off value of 7 g/dL [49]. Although the Frankfurt Consensus Conference recommends that haemoglobin concentration alone should not determine the need for red blood cell transfusion [29], patients with acute blood loss could benefit from transfusion even with higher Hb values. The precise indications for transfusion remain the subject of debate, which explains the variability in clinical practice [34]. In relation to FFP, the main indication for plasma transfusion is to correct deficiencies of coagulation factors, a blood component prepared from whole blood or collected by apheresis, frozen within time limits. The FFP contains normal levels of stable coagulation factors, albumin, and immunoglobulins with at least 70% of the original coagulating factor VIII and at least similar amounts of other labile coagulation factors and natural coagulation inhibitors. Process-wise, the FFP must thaw between 30 °C and 37 °C in a water bath with continuous agitation or with another system capable of ensuring a controlled temperature. Plasma should be transfused as soon as possible after thawing, but in any case, within 24 h, if stored at 4 ± 2 °C; FFP should not be refrozen once thawed. The recommended therapeutic dose of FFP is 10–15 mL/kg body weight. However, the dose of FFP depends on the clinical situation and laboratory parameters. The American Association of Blood Banks recommends transfusing hospitalized adult patients with a platelet count of 10 × 10^9^ cells/L or less to reduce the risk of spontaneous bleeding. The American Association in Blood Banks recommends transfusing up to a single apheresis unit or equivalent. Higher doses are no more effective, and lower doses equivalent to half a standard apheresis unit are equally effective. In addition, platelet transfusion is considered as an additional measure to those suggested above for critical bleeding. We should also consider the administration of tranexamic acid in trauma patients who are bleeding, surgical patients who are expected to have a blood loss greater than 500 mL. or bleeding associated with major trauma, and administer fibrinogen (concentrate or cryoprecipitate) if plasma fibrinogen concentration is <1.5 g/L or if signs of functional fibrinogen deficiency are seen on near-patient testing [49,50,51,52].

In summary, our study has shown the epidemiological characteristics of transfusion in trauma patients in 111 Spanish ICUs. Transfusion was found to be practiced with RBC and FFP platelets in a ratio of 2:1:1. Analytical results together with anaemia and haemodynamic instability are the main reasons for transfusion. The practice of transfusion does not vary depending on patient type, attending instead to the patient’s clinical situation. In non-polytraumatized patients, transfusion seems to be more effective in raising Hb levels.

### Limitations

The cross-sectional design of this study limits results in so far as there is no patient follow-up, and therefore the results obtained in relation to mortality cannot be compared with other longitudinal studies. However, based on the results obtained, a registry has been created to be able to follow the evolution of these trauma patients admitted to the ICU. We can thus analyse mortality and the appearance of complications related to blood transfusion; the characteristics of blood components, age, red blood cells, and platelets are not collected. On the other hand, the number of subjects included in the final sample is limited considering the inclusion criteria and it is, therefore, necessary to continue with new studies with larger samples to confirm the results obtained. In addition, the gender bias found in our study should be further studied in order to assess the effect of gender on the transfusion decision-making for trauma patients.

## 5. Conclusions

The results of our study demonstrated that most Spanish ICUs had implemented MTPs; however, it is necessary to continue implementing this type of programme in the hospitals that have not yet developed it. Similarly, we conclude that it is important to know the epidemiology of transfusion practices and to implement massive transfusion protocols in order to improve outcomes. The patient management programs currently are the best programmes to improve the ratio of transfusion. Although with great caution, given the design of our study and a lack of a longer patient follow-up, our study suggests that the transfusion of RBC, platelets, and FFC in a 2:1:1 ratio could be effective in decreasing the mortality rates of severe trauma patients.

## Figures and Tables

**Table 1:** Demographic and Clinical Characteristics of the Sample. Polytrauma patients; rest of patients.

		Polytrauma Patients		Rest of Patients		*p*-Value
		(*n* = 109)		(*n* = 1330)		
		Frequency	%	Frequency	%	
Age (mean ± SD)		41.42 ± 18.82		62.61 ± 15.06		<0.001
Gender	Men	93	85.32	858	64.56	<0.001
	Women	16	14.68	471	35.44	
SOFA-score (mean ± SD)		5.04 ± 3.46		4.71 ± 3.87		0.406
ICU stay (mean ± SD)		13.31 ± 20.53		10.40 ± 17.25		0.092
Mechanical ventilation	No	45	41.28	714	53.81	0.012
	Yes	64	58.72	613	46.19	
Extrarenal purification	No	107	98.17	1209	91.04	0.010
	Yes	2	1.83	119	8.96	
Anticoagulated patient	No	102	94.44	1056	79.94	<0.001
	Yes	6	5.56	265	20.06	
Anti-aggregate patient	No	101	92.66	1019	76.79	<0.001
	Yes	8	7.34	308	23.21	
ECMO *	No	107	98.17	1313	99.09	0.983
	Yes	2	1.83	12	0.91	
Transfusion	No	92	84.40	1201	90.50	0.041
	Yes	17	15.60	126	9.50	

* Extracorporeal membrane oxygenation.

**Table 2:** Description of the ICUs participating in the study.

Variable Frequency (*n*)		Percentage (%)	
**Type of hospital**			
	Public	109	98
	Private	2	2
**Bed numbers in the hospital**			
	<500	34	31
	500–1000	44	40
	>1000	33	30
* ICU’s Bed numbers		23.41 ± 10.31 (6–48)	
**Transfusion protocol**			
	No	43	39
	Yes	68	61
**Massive transfusion protocol**			
	No	24	22
	Yes	87	78
* PBM programme			
	No	72	65
	Yes	39	35

* ICU’s = Intensive Care Unit’s beds; PBM = Patient Blood Management.

**Table 3:** Demographic and Clinical Characteristics on transfused patients.

		Polytrauma Patients		Rest of Patients		*p*-Value
		Frequency	%	Frequency	%	
Age (mean ± SD)		39.00 ± 19.67		66.00 ± 14.37		<0.001
Gender	Men	11	64.71	87	69.05	0.717
	Women	6	35.29	30	30.95	
SOFA-score (mean ± SD)		7.00 ± 3.75		7,00 ± 3.95		0.424
* ICU stay (mean ± SD)		5.00 ± 28.95		5.50 ± 14.37		0.764
Mechanical ventilation	No	3	17.65	44	34.92	0.155
	Yes	14	82.35	82	65.08	
Extrarenal purification	No	16	94.12	100	79.37	0.145
	Yes	1	5.88	26	20.63	
Anticoagulated patient	No	16	94.12	89	72.36	0.052
	Yes	1	5.88	34	27.64	
Anti-aggregate patient	No	13	76.47	101	80.16	0.723
	Yes	4	23.53	25	19.84	
* ECMO	No	17	100.00	121	96.03	0.403
	Yes	0	0.00	5	3.97	

* ICU = Intensive Care Units; ECMO = Extracorporeal membrane oxygenation.

**Table 4:** Transfusion practice description.

Transfusion		Polytrauma Patients		Rest of Patients		*p*-Value
		(*n* = 17)		(*n* = 126)		
		Frequency	%	Frequency	%	
Reason for transfusion	Acute anaemia with haemodynamic impact	3	17.65%	44	34.92%	0.582
	Acute anaemia without haemodynamic impact	3	17.65%	27	21.43%	
	By analytical criteria	7	41.18%	45	35.71%	
	Previous intervention or invasive procedure	2	11.76%	9	7.14%	
	Others	2	11.76%	9	7.14%	
Blood component	Red blood cells concentrate	15	88.24%	113	89.68%	0.736
	Fresh frozen plasma	7	41.18%	48	38.10%	0.940
	Platelets	7	41.18%	50	39.68%	0.848
Number of transfusion events	1	7	41.18%	100	79.37%	0.707
	2	6	35.29%	13	10.32%	
	3	1	5.88%	8	6.35%	
	4	2	11.76%	2	1.59%	
	5 or more	1	5.88%	3	2.38%	
Transfusion control	No	3	17.65%	18	14.29%	0.481
	Yes, in 1 h	1	5.88%	11	8.73%	
	Yes, in 2 h	5	29.41%	16	12.70%	
	Yes, in 3 h	2	11.76%	19	15.08%	
	Yes, in more than 3 h	6	35.29%	62	49.21%	

**Table 5:** Difference between pre-transfusion and post-transfusion blood levels.

Transfused Patient Previous		Post-Transfusion			*p*-Value			
		Median	P25	P75	Median	P25	P75	
Polytrauma patient	haemoglobin	8.6	8.1	10.4	9.65	8.6	10.75	0.077
	MCV	91.2	87.7	95	91.8	87.9	92.5	0.979
	INR	1.13	1	1.2	1.1	1	1.27	0.353
	platelets	151	129	246	183	122	224	0.988
Other patient	haemoglobin	7.6	6.9	8.6	9	8.4	10	<0.001
	MCV	90	86.6	95	90	85.7	93.5	0.004
	INR	1.2	1.08	1.5	1.22	1.09	1.42	0.055
	platelets	148	78	220	137	79	192	0.990

**Table 6:** Logistic regression to identify factors associated with the transfusion of blood components.

Risk Factor OR	*p*-Value	IC95%	Inferior	Superior
Trauma	1.75	0.049	1.01	3.36
Anticoagulated	1.54	0.045	1.00	2.43
SOFA	1.13	0.000	1.08	1.18
VM	1.58	0.037	1.03	2.44
Haemoglobin previous	0.89	0.008	0.82	0.97
Hosmer–Lemeshow *p*-value			0.839	
C-statistic			0.787	

## Data Availability

The anonymized data presented in this study are available at the request of the last-mentioned author. The data are not publicly available due to current personal data protection legislation.
